# What Can Be Learned from Experience with Scientific Advisory Committees in the Field of International Environmental Politics?

**DOI:** 10.1002/gch2.201800055

**Published:** 2018-08-05

**Authors:** Steinar Andresen, Prativa Baral, Steven J. Hoffman, Patrick Fafard

**Affiliations:** ^1^ Fridtjof Nansen Institute 1366 Lysaker Norway; ^2^ Global Strategy Lab York University/University of Ottawa Canada; ^3^ Dahdaleh Institute for Global Health Research Faculty of Health and Osgoode Hall Law School York University Toronto Ontario Canada; ^4^ Graduate School of Public & International Affairs University of Ottawa Ottawa Ontario Canada

**Keywords:** environmental regimes, global environmental policy, international environmental politics, science–policy nexus, scientific advisory committees

## Abstract

Scientific advisory committees (SACs) are a critically important part of global environmental policy. This commentary reviews the role of SACs in six global and regional environmental regimes, defined here as the set of rules, norms, and procedures that are developed by states and international organizations out of their common concerns and used to organize common activities. First, SACs play a critical role in putting issues on the political agenda and the creation of an overarching regime. Second, the effectiveness of a given SAC and the associated regime is highly variable. Third, there is also considerable variation in the extent to which the regime is driven by an overarching scientific consensus, for example, high in the case of climate change, lower in the case of whaling. Fourth, the role of science in a given regime is also a function of whether the problem being addressed is relatively benign or more malign, that is to say, marked by deep political disagreements (i.e., climate change). Finally, the cases examined here suggest that the institutional design of the SAC matters and can influence the overall effectiveness of the SAC and by extension, the regime, but it is seldom decisive.

## 1 Introduction

The role and significance of science has been questioned globally for decades by a myriad of scholars and policymakers alike. The Scientific Committee of the International Whaling Commission (IWC) is but one example of scholars coming together to form a global scientific advisory committee (SAC), in part, to encourage the role of science in promoting an orderly development of the whaling industry.^1^ Still, the level of involvement of science is often unclear, particularly so in the field of international environmental politics, where competing stakeholders and interests exist simultaneously.

In the study of international relations theory, we define a regime as the set of rules, norms, and procedures that are developed by states and international organizations out of their common concerns and are used to organize common activities. Thus, a regime is much broader than a SAC but it is likely these committees will be an integral part of a given regime, particularly in areas like health and environment where scientific research is critically important to establishing the agreed‐upon rules and procedures.^2^ In 2000, Andresen et al. published a book where international environmental regimes were analyzed, with the aim of better understanding the role of science in the operation, management and overall effectiveness of these bodies. These five regimes, notably the IWC, the global UN climate regime, the global ozone regime, the North Sea environmental regime and the (mostly) European acid rain regime, were chosen as they best exemplified the layers of global and regional complexity involved in this dialogue. Ultimately, the book analyzed the extent to which scientific advice was followed by relevant policy makers and contributed to the overall effectiveness of the regimes, with a particular focus on the significance of the science–policy designs.^3^

The theoretical groundwork provided by Underdal in the book has been a noteworthy analytical point of reference for many of the subsequent works on the science—policy nexus in international environmental policies. Since its release, additional publications have delved into new important areas and regimes such as technology and biodiversity, respectively. The empirical data have also been updated since then, leading to the creation and discussion of novel theoretical approaches. The purpose of this commentary is nevertheless not an exercise in the synthesis of these new findings; rather, we hope to provide some useful lessons for current and emerging international health regimes to maximize their overall impact.

At the outset, the most important and sobering lesson to note is the following: the role and influence of science as well that as of various scientific advisory committees should not be overtly exaggerated. This is because scientific evidence is but one of several legitimate premises that policy makers need to consider during the decision‐making process. Realistically, more weight is usually attributed toward economic and political considerations. Simply put, politics is very rarely driven by science alone. Clever institutional designs of scientific advisory committees may make a difference in terms of the extent of the influence they hold but this is not always the case. Often, their influence (or lack thereof) is contingent on the nature of the issue being scrutinized. That is, the higher the intensity of political conflicts and scientific uncertainty, the less likely it is that scientific advice will be adhered to—and vice versa.^4^

With this caveat, we have organized this article as follows. First, we review various approaches to understanding the role of science in policy‐making as well as the relationship between science and the effectiveness of these international regimes. Next, we examine the establishment of international environmental agreements and associated scientific committees: what actors and interests are needed for them to emerge? Last, we reflect on the lessons that can be learned with regards to their role and influence.

Empirical examples are mainly drawn from the global regimes studied; considering the high number of participants and the (usually) strongly divergent interests, challenges of reaching effective solutions are most severe here. The two regional regimes will also be addressed to a lesser extent.

## 2 The Role of Scientific Expertise: Various Approaches

There are various approaches to the study of the science–policy nexus. One such method is to elevate the status and the influence of science by making it appear more legitimate via a broad participatory approach. This strand of research claims that the necessary knowledge needed to secure “good governance” goes beyond the stipulations of the traditional and narrower scientific community. In order to achieve this, they recommend diversifying the key stakeholders by inviting private actors and members of the broader public in the assessments of a variety of scientific disciplines. This approach asks that both normative and political elements be accepted in the decision‐making process.^5^

This all‐inclusive approach is quite frequently applied at the local level but presents logistical challenges in its application at the international level. For example, even when only traditional scientists are involved, there may be hundreds or thousands represented at various stages of the decision‐making process, as seen in the International Panel on Climate Change (IPCC).^6^ Adding stakeholders from other fields and areas of expertise would almost certainly contribute to a vast and unruly process that would be challenging to organize and govern. It is our view that that such processes should therefore be left to scientists, with the additional stakeholders contributing their interests through lobbying and other mechanisms at later stages. While one partial exception to this is the recent establishment of the International Panel on Biodiversity and Ecosystem Services (IPBES), it is too soon to depict its effectiveness long‐term (Andresen and Rosendal, 2017).^7^

Another approach and associated discussion (and controversy) relates to the extent to which science should be independent or embedded in the political process. The traditional position has always been one where science is seen through a value‐free and objective lens, coined by the phrase, “Speaking truth to power.”^8^ Yet some argue that such objectivity is neither possible nor should it be the ultimate goal.^5^ We agree that objectivity in its strictest sense is difficult to achieve, if not impossible to obtain, particularly given the variability of the disciplines involved.

There has been much debate concerning the fruitfulness (or lack thereof) of separating the “scientific” from the “political” sphere.^8^ Though this is an important debate, there are no easy answers. In an effort to simplify this debate, we have purposefully chosen to take a simple point of departure throughout the article. Based on lessons learned from these international regimes, and, in line with the rationalist tradition, we project that knowledge produced by various groups of scientists is a necessary but insufficient condition for designing effective international SACs. That is, the most important task of scientists has been to warn policymakers of the endangered environmental and natural resources, should present policies be continued. To illustrate, we ask that you reflect for a moment on the state of the art, in the absence of science. Recall the classic metaphor of the “tragedy of the commons,” where short‐sighted economic interests tend to prevail over concerns regarding the common good. However imperfect, we argue that scientific input is needed to balance these competing forces. Without sound evidence or good knowledge, management of the environment will tend to be based either on luck or exclusively on economic and political priorities.

What is the conceptual relationship between scientific input and the effectiveness of regimes? International relations scholars have studied this connection for more than two decades.^4^ A number of intricate methodological challenges have been discussed, but for the purpose of this article, we have chosen to only address briefly how effectiveness can be explained. As indicated in the introductory paragraph, the most important explanatory determinant is the political nature of the issue at stake, that is, how politically and intellectually “malign” or “benign” the problem is.^4^ The more “malign” an issue‐area is, the more difficult it can be to deal effectively with the problem. In short, the political dimension seems to be a much more important factor and hold more clout than the intellectual one: that is, when political conflicts are strong (i.e., malign), even if the intellectual dimension is benign via scientific consensus, effectiveness tends to be low (Andresen, 2013).^9^ As a result, high effectiveness of regimes is associated with low political conflicts and high scientific certainty. Alternatively, while this benign versus malign structure of a problem can be considered to be external to the regime, an internal regime‐specific explanatory factor deals with the regime's specific problem‐solving ability: how effective is the regime in solving the challenges it is set up to deal with? This ability to solve problems can be seen as a function of the leadership and skills of the participating countries, as well as the institutional structure of the regime itself. If the leadership is exerted by powerful actors within an advanced institutional structure that is procedurally sophisticated for science–policy interactions, the effectiveness of the regime will justifiably be enhanced. In other words, some problems are attacked with more political and institutional energy than others.

Scientific expertise is an important factor that determines both the nature of the problem and the strength of the problem‐solving capacity of a regime. Regarding the nature of the problem, the simple assumption is that, all things being equal, the higher our knowledge and consensus on a topic, the higher the likelihood of a regime with increased effectiveness. This subsequently impacts the problem‐solving capacity of a regime through its involvement in the design of the science–policy nexus and the question of independently developed research as compared to those embedded in politics. Our rationalist and positivist approach implies a highly independent approach to science, but when confronted with the highly contested political realities globally, this is too simplistic. There is a need to create a balance between scientific integrity and political involvement to ensure that scientific experts recognize the needs of decision‐makers and present legitimate scientific results that can be utilized effectively in practice.^3^

## 3 The Establishment of Scientific Advisory Committees

What are the role and responsibilities of scientists and of other actors in communicating pressing problems to policymakers, setting agendas, and developing international committees?

Based on previous experiences in international environmental affairs, the short answer is that the role of scientists is extremely crucial. First, had it not been for the “whistle‐blowing” from the scientific community, many environmental problems would not have been acknowledged until it was long overdue. Second, the amount of time elapsed between the initial identification of the problem by scientists and its subsequent inclusion in policy makers' agenda is considerable. Third, in order for action to be taken, recommendations and warnings given by scientists need support from other stakeholders. For example, environmental NGOs are known to be experts in simplifying and amplifying often complicated “ivory‐tower” academic research. Similarly, international organizations and key states can be powerful contributors in paving the ground for political action. Fourth, “external shocks” and strong media exposure may also spur regime creation.

### 3.1 International Regimes

We will illustrate these general points by relying on some examples of international regimes. Global warming was first brought to attention by US scientists in the late 1950s and for more than two decades it remained almost exclusively within the sphere of various international scientific committees. It only reached the international political agenda in the mid‐1980s through the establishment of the IPCC in 1988. Key players such as the United Nations Environmental Program (UNEP), the World Meteorological Organization (WMO), green NGOs, activist policy‐makers, and scientists were all instrumental in drawing attention to this issue. The United States, with its dominant research experience in the field combined with its political clout, played a crucial role in launching the IPCC.^10^ In this particular example, the scientific advisory body preceded the political negotiations, which began in 1989 (Young and Osherenko, 1993;^11^ Agrawala and Andresen, 2002).^12^

Ozone depletion was another major environmental issue brought to the forefront in 1974 by US scientists. The WMO and the UNEP were also important actors in drawing attention to this problem. As a result, political negotiations began in 1981, and the Vienna Convention was adopted four years later (Benedick, 1998).^13^ The scientific discovery of the “ozone hole” above Antarctica in 1985 accelerated the political process toward the stricter regulations of the Montreal Protocol in 1987, in part due to the massive attention placed by media on the potential environmental and health threats associated with this hole. As a result of this “external shock,” scientific expert panels were set up, leading to the adoption of the Protocol. A year later, in 1988, scientific findings concluded that there were indeed strong indications to associate man‐made ozone‐depleting gases as the leading cause behind the observed thinning of the ozone layer. In this example, the US was not only a scientific leader, but also initiated the political process to act rapidly.^14^

In the case of the whaling issue, the newly established International Council for the Exploration of the Seas (ICES) released warnings in 1900 regarding the overexploitation of large whales. However, whaling nations ignored ICES's counsel and whaling increased steadily. Alarm bells from scientists alone are therefore not sufficient to trigger policy action or the creation of regimes, particularly when strong economic interests are at stake.^15^ Attempts to create international regulations emerged in 1930s but this had no demonstrable impact with regards to the catch. Furthermore, there was no scientific clout placed behind these efforts. It was only post‐World War II that this changed, with the creation of the International Whaling Convention (1946) and the subsequent International Whaling Commission 1948. The role of science figured prominently in the Whaling Convention, in part due to the role of US biologist Brian Kellog, and a Scientific Committee was finally established in 1951.^1^

What was the reason behind the delay in the establishment of a permanent scientific panel for the biodiversity convention? One reason may simply be a lack of scientific uncertainty and disagreement on this issue. While some uncertainty exists with regards to estimating the number of species disappearing, there is practically no scientific discord concerning the severity of the loss of biodiversity. This is in part due to a number of already existing key scientific advisory committees, all agreeing on the severity of the problem (Andresen and Rosendal, 2017). Another explanation is the instrumental role of the UN Millennium Ecosystem Assessment in providing rigorous scientific advice. However, the main reason behind the delay in establishing a permanent panel was probably due to a lack of powerful stakeholders championing such a body, in contrast to ozone depletion and global warming. The USA as well and other developing states, for various reasons, saw no need for such an institution (Andresen and Rosendal 2017).

### 3.2 Regional Regimes

Scientists from Sweden and Norway, some of the most negatively affected states, were instrumental in setting the agenda for the acid rain regime. By sounding the alarm on their respective governments, they were instrumental in rapidly setting up the regime in 1979.^16^ In comparison, scientists had minimal role in the establishment of the North Sea regime. Instead, strong media attention over incidents of hazardous waste dumping triggered a public outcry similar to an external shock: by the late 1960s, companies were dumping toxic waste in the North Sea and no regulations existed. This praxis spurred the subsequent political action, leading to the establishment of the North Sea environmental regime in 1972.^17^

## 4 The Influence of Science

### 4.1 How Effective are the Regimes?

Before exploring how influential science has been on adopted policies, we should address the effectiveness of these regimes in managing the problems they have been designed to address. Previous research indicates that the ozone regime is a rare success story in international environmental politics, as ozone depletion no longer presents an imminent threat to the environment (Miles et al. 2002;^4^ Andresen et al. 2012).^18^ In contrast, both the biodiversity and the climate regimes represent an alternative picture at the other end of the effectiveness spectrum. The main purpose behind the climate regime was to reduce greenhouse gas emissions, but since its establishment in 1992, emissions have increased steadily. Similarly, while the biodiversity regime was primarily designed to halt biodiversity loss, this loss has continued unabated and has amplified since its establishment. Thus, from a problem‐solving perspective, they are not being effective.

In contrast, the whaling regime is more challenging and controversial to discuss in terms of effectiveness. If the political will of the anti‐whaling majority of the IWC members is considered to be the benchmark for effectiveness, the score is indeed high, as commercial whaling has been banned since 1982. However, if the main goal of the Whaling Convention is to secure the orderly development of the whaling industry through scientific evidence‐based decisions as laid down in the International Whaling Convention, the score is low. The whaling industry has been wiped out and scientific advice is unfortunately not adhered to. This will be elaborated further below.

The overall effectiveness of the two regional regimes has been somewhat higher, in part attributed to the fewer and more homogenous actors involved, which may have contributed to fewer political conflicts. This has also contributed to more extensive adherence to scientific advice over time.^4^, ^14^, ^16^, ^19^

### 4.2 Consensus in Science is Usually Accepted—But Not Always Applied

Although these examples illustrate the significant variation in the effectiveness of regimes, there has been a general inclination toward additional scientific input and greater formalization of the relationship between decision‐making bodies and the scientific community over time. The establishment of the Scientific Committee of the IWC was a slow process and even then, very few scientists were involved during its first two decades. In more recent international regimes, scientific bodies are set up concomitantly with—or even prior to—the establishment of international political bodies, as witnessed in both the climate and the ozone regimes. There has been an observable increased affinity toward requesting a broad variety of scientific inputs.^3^, ^20^ For example, while biologists have long dominated agenda setting in the IWC Scientific Committee, input from statisticians was necessary to use large computer models to assess the size of various whale stocks. Similarly, contributions from economics as well as other social scientists are now frequently included in scientific assessments, not the least in the IPCC.

In the early stages of negotiations when the strength of the scientific evidence was weak or disputed, this uncertainty was frequently exploited to serve political and economic interests. Progress has often been hampered by one or more parties demanding more conclusive evidence, or by providing self‐serving interpretation of available information. A typical example of this was observed in the United Kingdom during the early phase of both the acid rain and the North Sea regimes: the UK claimed that scientific evidence was inconclusive and as such, no action was necessary. And yet, the effectiveness for both the acid rain and the North Sea regimes has increased since then. A contributing factor is likely to have been the growth in scientific consensus since the early phases of the regimes.^16^, ^17^ With regards to the issue of climate change, most countries accept the recommendations presented by the IPCC and are taking tangible measures to address the problem. However, these have not been sufficient to reduce emissions. Previously, although President Bush had rejected the Kyoto Protocol, he had accepted the scientific underpinning of the problem: he chose an approach that preferred voluntary control measures to reduce emissions as opposed to a mandatory ceiling. However, it appears that Donald Trump, the current president of the United States, has chosen to reject the scientific consensus from the IPCC altogether. Similarly, the IWC rejects the scientific opinion of the large majority of scientists advocating for sustainable commercial whaling practices. The lack of scientific influence here is in part due to the strong positions taken by a handful of antiwhaling scientists and because values tend to dominate political discussions. As a result, there is little room for scientific influence when the main discourse is strictly between pro—or antiwhaling.^20^

On the other hand, the biodiversity and the ozone regimes demonstrate a long‐standing scientific consensus with regards to their respective issues, and thus, a lack of political controversy. And yet, while policy‐makers in the ozone regime follow the recommended scientific advice, the opposite is observed in the biodiversity regime. This may be explained to some extent by the presence of highly advanced scientific advisory bodies in the ozone regime whereas the IPBES of the biodiversity regime was but recently established. But the most important reason is no doubt that the biodiversity case is much more of a politically malign nature, as compared to the challenges faced by the crafters of the ozone regime.

Based on the case studies explored here, we argue that generally speaking, the pattern is one where broad scientific consensus about a problem tends to facilitate problem‐solving, but this is primarily so regarding regimes facing fairly benign problems. As a result, some actions are usually taken based on scientific advice, but nevertheless, they still usually fall short of scientific recommendations. In some rare cases however, the regulatory body moves substantially beyond these recommendations: in 1982, the IWC put forward a ban on commercial whaling despite the scientific committee's recommendation for a more selective approach.^1^

### 4.3 Institutional Design Matters But Is Seldom Decisive

We have chosen to focus on the ozone and climate global regimes to illustrate the significant variation in their influence in policy‐making despite the sophisticated institutional design of their respective scientific advisory committees.

The ozone regime has three scientific assessment panels: these pertain to science, environmental effects and technology, and economy. Since the first set of assessment publications in 1989, the three panels have published periodic evaluations in their respective fields every four years. The key findings of the panels for each periodic assessment are synthesized into a short report.

This institutional framework represented a formalization of the science‐policy nexus that had previously appeared solely in the United States. Now, the ozone regime was formally introduced and subordinated to the political and regulatory bodies, broadening the interests it represented. Today, although the regime still remains formally under political control, the advisory bodies have continued to maintain both scientific authority and political impartiality. Agreement between both policy‐makers and analysts of this regime indicate that these scientific advisory panels have always represented key pillars of the ozone protection regime, from the initial implementation of the Montreal Protocol to the present.^21^ In addition, there is no doubt that the recommendations from the panels have been instrumental in the significant increase in the strength of the rules and regulations of this regime. Moreover, these scientific panels cover all issue‐areas relevant for effective regulation of ozone depleting substances, including economic interests and technological developments, thereby moving significantly beyond the narrow definition of “science” that had dominated prior to their establishment. As a result, members of the panels have high levels of expertise and scientific credentials, but provisions nevertheless exist to secure a regional balance.^21^

We therefore argue that the role of the scientific advisory body and the design of the science–policy interface can be a factor in explaining the effectiveness of this regime. To illustrate, policy‐makers would not have had the tools and mechanisms to tailor and design the measures effectively without the formalization of the elaborate three‐panel scientific apparatus in place. Due to the scientific consensus reached by these panels, the nature of the problem was classified as being benign, increasing the regime's effectiveness. Moreover, the problem‐solving ability of the regime has also been strengthened by the organization of this science‐policy nexus: in contrast to the other regimes studied here, it therefore appears that regulations adopted by this regime are driven by science.

There nevertheless are important caveats to this observation. The likelihood of these policies being adopted would be low had it not been for the interaction of scientific advice and institutional design with other factors that helped promote the aggressive antiozone depletion measures taken. That is, the consensual science and the well‐organized science–policy nexus of the ozone regime would not have been sufficient alone. First, the gradual expansion of states' participation in the regime was one factor that influenced the regime's effectiveness. Initially, when only a few states with vested interests participated in these panels, consensus on key issues could be more easily reached and the influx of newcomers tended to accept the scientific message already reached. At present, it is truly a global regime with the participation of more than 190 states. Second, developing countries were given a grace period of 10 years to begin the enforcement of the commitments and regulations, giving them additional opportunities to meet the regime's goals. Third, the establishment of the Multilateral Fund has been highly effective in assisting developing countries in meeting these recommendations. The leadership demonstrated by the United States, one of the world's most powerful nations, in promoting strong regulations has also contributed significantly towards the high effectiveness of this regime.^14^

There are also key intrinsic features of ozone depletion that can explain the successful regime. First, the problem became more manageable than other global environmental challenges due to the relatively few producers and consumers of ozone depleting substances. Second, and perhaps most important for the effectiveness of the regime, available substitutions to the harmful substances used were easily available, thereby providing a relatively “quick technology fix” to the problem. In short, these factors made it such that ozone depletion was characterized as a benign problem.^20^

With regards to the climate regime, as previously noted, the IPCC was established prior to the start of political negotiations. The publication of the First Assessment Report [by the IPCC?] in 1990 may have been a contributing factor to the adoption of the Climate Convention soon afterwards in 1992. To note, the IPCC's design features include a variety of different task forces, a secretariat and three working groups focused on science, impact and response strategies respectively. The working groups publish full reports and summaries, which subsequently provide the framework for the synthesis report. The entire IPCC meets periodically to approve these reports, prior to their release. Finally, government representatives conduct a detailed review of the summary for policymakers. This demonstrates the tight political control in the final stage of this process.^6^

With the exception of the final stage, however, the process is characterized by scientific independence and a thorough review process. Lead authors prepare the first drafts with contributing authors providing assistance in special sections. The draft reports then undergo two rounds of scientific review to ensure consensus. In recent years, thousands of experts from more than 130 countries have contributed in various capacities. Since its first publication in 1990, the IPCC has released subsequent Assessment Reports in 1993, 2001, 2007, and 2013.

The credibility, relevance, and international platform of the IPCC were demonstrated in 2007 when it was awarded the Nobel Peace Prize (together with Al Gore). Nevertheless, although its message has become increasingly alarmist and unified over time, climate change skeptics from various camps have continued to distrust the IPCC. This was amplified by the so‐called “Climategate” in 2009, where it became known that renowned climate scientists had attempted to minimize the influence of critical views and failed to disclose their disputes.^3^, ^4^, ^20^ Moreover, the prediction contained in the 2007 IPCC Report stating that the Himalayan Mountains would lose all of their glaciers within 25 years proved to be inaccurate. These events prompted several internal changes to IPCC procedures, including the establishment of a task force to restore its credibility.^18^ There were no major controversies in the aftermath of the release of the 2013 IPCC Report; it appears that the main deficiencies have been resolved. This illustrates that constructive criticism from both the media and the policy‐makers is sometimes necessary for the elaboration and organization of scientific advisory panels. That is, this interaction between SACs and policy‐makers is a two‐way street.

Overall, it is apparent that the IPCC has provided solid scientific recommendations to policy‐makers. Communication with the broader public has also improved significantly over time. While one of IPCC's key features is its intergovernmental nature, there has been criticism that its influence may be reduced given that its scientific independence can be questioned.^8^ Conversely, most analysts claim that the intergovernmental nature has strengthened the role of the IPCC by increasing its legitimacy and relevance.^5^, ^22^ The key argument is that political involvement and scientific autonomy serve different functions at different stages of the science‐policy nexus. Autonomy is necessary for producing science, whereas involvement is essential for transforming science into policy‐relevant scientific input. Moreover, autonomy and involvement have the potential to serve different roles based on the parties involved. For OECD countries, the autonomy of science is a core value. From the perspective of the Global South, “autonomous” science is often perceived as science done by the (rich) North, for the North and as such, they ask for political control in the latter stage of the process. The overall “institutional design” lesson is that some political control over the scientific process is required to secure the legitimacy of scientific expertise in highly contested global regimes.^3^

The IPCC has significantly contributed to the reduction of scientific uncertainty, and has thereby reduced the complexity of the nature of the environmental problem to be solved. The design of the science–policy intersection has also contributed in increasing the problem‐solving ability of the regime. Thus, using the counterfactual argument, were the IPCC nonexistent, the climate change regime would in all probability have scored (even) lower in terms of effectiveness. But why is the climate change regime still deemed to be ineffective? The primary reason lies in the malign nature of the problem of climate change when compared to most other environmental issues. That is, the climate change problem is much more than an environmental problem alone, as it affects the economy, the trade, the energy and the development of countries significantly. Vested interests and conflict between nations makes political action very challenging. The problem‐solving ability of the regime has also been hampered by conflicting choices in the approach to take as well as the mostly negative stance taken by the US in the process. Still, there is no doubt that the IPCC has contributed significantly to an increased understanding among the public about the severity of this complex problem.^18^ Furthermore, in the aftermath of the ambitious goals set by the 2015 Paris Agreement, it may be that policy‐makers are finally paying attention to the warnings from this scientific advisory committee. Whether this materializes into tangible action on the ground however, remains to be seen.

## 5 Concluding Comments

Some of the conditions needed for scientific expertise to influence decision‐makers and enhance the effectiveness of international environmental regimes are addressed in this commentary. First, scientists are crucial in the setting of agendas: their scientific discoveries are after all the core upon which frameworks can be created to confront environmental challenges. However, the creation of regimes requires active efforts from other stakeholders such as environmental groups, international organizations as well as powerful states. The conditions under which scientific advisory committees can influence policy‐makers and contribute to more effective international environmental regimes are several. Scientific expertise represents an important premise for decision‐makers in international environmental regimes, as scientific bodies are generally included in their institutional framework. The increased range of scientific input introduced in the regimes is another important factor. In addition, governments rarely dispute scientific consensus, which typically leads to some level of subsequent collective action to address the environmental concern. However, action alone is very seldom sufficient to solve the problem at hand.

Generally speaking, scientific expertise seems to be most important in the agenda‐setting phase, and gradually declines in significance as politics take control over the relevant decision‐making processes. This is particularly true in global regimes such as biodiversity and climate change with highly complex political problems. The political action taken in these two regimes to this point has been too weak to effectively solve the problems they were designed to address. Given that political interests are usually deemed to be more influential than scientific input, it is clear that scientific advisory committees alone are intended to be but one contribution in the larger dialogue. Still, using the counterfactual, effectiveness would have likely been even more modest in the complete absence of scientific input. The ozone regime is the only global regime in our sample where decisions followed scientific advice. However, this was primarily due to the presence of other contextual factors aligning toward the direction paved by the scientific counsel. In contrast, the majority of IWC members reject the advice from the overwhelming majority of scientists in the Scientific Committee on the crucial issue of commercial whale catching practices: this is due to conflicts over value articulation which differ vastly at the global scale. That is, when there are strong conflicts over values, room for rational input represented by science tends to be small.

Finally, with regards to the design of the science–policy nexus, in highly contested global regimes like climate change, experience indicates a need to strike a balance between scientific autonomy in the production of knowledge and some degree of political control in the finalization of the policy making process in order to reach scientific consensus. This may be one of the main reasons why the more recent IPBES for the biodiversity regime has replicated the intergovernmental approach of the IPCC. In contrast, where political conflicts feature less prominently, such as the case of the ozone regime, more autonomy is given to the scientific advisory bodies.

To conclude, we argue that scientific advisory committees are but one variable in explaining the effectiveness of international regimes. However, these expert committees serve more than one purpose. Beyond simply informing a decision, SACs can help set an agenda, emphasize a particular value articulation, and promote stakeholder engagement to address environmental challenges with added clout.

## Conflict of Interest

The authors declare no conflict of interest.
